# Patient-related factors may influence nursing perception of sleep in the Intensive Care Unit

**DOI:** 10.1371/journal.pone.0226323

**Published:** 2020-01-06

**Authors:** Mariam Louis, Kasey Treger, Tracy Ashby, Carmen Smotherman, Shiva Gautum, Vandana Seeram, James Cury, Lisa Jones

**Affiliations:** University of Florida, Jacksonville, UF Health Clinical Center, Jacksonville, Florida, United States of America; University of Rome Tor Vergata, ITALY

## Abstract

**Objective:**

There exist conflicting data regarding the accuracy of ICU nurses accurately assessing patient sleep using validated questionnaires. Using the Richards-Campbell Sleep Questionnaire (RSQ), we hypothesize that patient factors might influence nursing perception of their sleep.

**Methods:**

Patients in the ICU who met the inclusion criteria were asked to complete the sleep questionnaire, as were their nurses and intraclass correlation analysis was done.

**Results:**

38 paired patient-nurse surveys were included for analysis. The mean difference in total average score of the RSQ was not significantly different between patients and nurses. There was fair intraclass correlation by patient age, black race, and admission for respiratory illnesses. A good intraclass correlation existed for non-blacks and admission for non-respiratory reasons. Most striking was the intraclass correlation by sex, with poor intraclass correlation for women compared to an excellent correlation for men.

**Conclusion:**

The results of our study confirm that patients in our ICU have poor sleep with a fair intraclass correlation. When examined by patient related factor, the greatest divergence between patient and nursing perception of sleep in the ICU using the RCSQ was patient female sex. More research is needed in this area to better understand the divergence and improve sleep in the ICU.

## Introduction

Among critically ill patients, it has been consistently reported that patients experience worse sleep quality in the Intensive Care Unit (ICU) as compared to home [1]. Poor sleep is an important source of ICU-related anxiety and stress amongst patients [2] and sleep is essential for energy conservation and cognitive function [3]. Disrupted sleep is associated with immune dysfunction, impaired resistance to infection, as well as alterations in nitrogen balance, wound healing and ICU delirium [4]. From prior studies, we know that sleep is generally light and fragmented among ICU patients [5, 6]. This can be attributed to both environmental and physiologic factors. Some environmental obstacles such as mechanical ventilation are essential to patient care and unavoidable. However, modifiable factors exist including noise, light, and timing of phlebotomy, imaging, and medications [3].

One aspect of sleep in the ICU less commonly studied are nurses’ perception of patient’s sleep. Nurses are at the forefront of patient care in the ICU, and can provide important information regarding the patient’s sleep. Although polysomnography remains the gold standard for assessment of sleep, its use in the ICU has some limitations including artifacts and divergence from normal sleep patterns [7]. Subjective survey instruments have been investigated as practical instruments to measure sleep in the ICU. The Richards-Campbell Sleep Questionnaire (RCSQ) is a visual analog scale that has been validated as an accurate assessment of sleep [8]. However, studies have been conflicting as to whether there is accurate patient-nurse interrater agreement, allowing nurses to complete RCSQ surveys in place of patients [9,10,11] Recently, a study published in Australian Critical Care comparing patients RCSQ score to nursing assessment and documentation found only a moderate agreement [12]. Furthermore, nurses’ perception of sleep has been studied and shown to have deficits in ability to accurately predict awakenings and sleep quality [12,13] and that nurses tended to overestimate patients’ perceived sleep quality [11].

To date however, patient related factors that may influence patient and nursing sleep perception have not been examined. Using the RCSQ score, we hypothesize that sex, age, race and reason for admission to the ICU influence sleep perception. We evaluated this through retrospective chart review of these groups of patients.

## Methods

### Participants

The study was conducted at the University of Florida, Jacksonville medical intensive care unit (MICU) between October 2014 and March 2015. Nurses had not received any prior education on sleep in the ICU, but were educated on identifying appropriate patients regarding inclusion and exclusion criteria for the questionnaire. Patients were identified by nursing staff and were included if they were older than 18 years old, capable of answering the questionnaire and had been admitted to the MICU for longer than 24 hours. Patients were excluded if they were sedated, deaf, blind, or unable to read and speak English, or if they had any obvious cognitive impairment or delirium.

### Procedure

Approval for the study was obtained from the University of Florida-Jacksonville Institutional Review Board. Both patients and nurses completed the RCSQ questionnaires regarding their sleep quality. The surveys were collected and a retrospective chart review of the medical record of those patients was then performed. The data collected included the variables age, sex, ethnic group and reason for admission.

### Data analysis

Descriptive summaries are frequencies and percentages for categorical variables and means, standard deviations (SD), and medians and ranges for numeric variables. The differences between patients and nurses survey scores were analyzed using paired *t*-tests. The intraclass correlation coefficients (ICC), along with their 95% confidence intervals (CI) were used as a measure of reliability of the patients and nurses responses. The level of significance was set at 5%. All analyses are performed using SAS ® Version 9.4 for Window.

## Results

A total of 128 of surveys were completed by both nurses and patients and 38 paired patient-nurse surveys were included for analysis. For duplicate surveys, the surveys completed on the first night in the ICU were included in the analysis. See S1 Fig for the flow diagram.

The patient demographic information is represented in Table 1. Sixteen (42%) of patients were male, and the median age was 55 years (range 22–83 years). Patient’s mean RCSQ scores ranged from 49.7 (on “Sleep depth” question) to 54.6 (on “Sleep latency” question, Table 2). The nurses’ scores ranged from 44.6 (on “Sleep depth” question) to 54.6 (on “Returning to sleep” question). The mean difference in total average score (2.4, 95% CI -4.9, 9.7) was not significant different between patients (mean 52.1, SD 24.9) and nurses (mean 49.7, SD 23.1). S2 Fig depicts the scatter plot for the paired patient-nurse total score on the Richards-Campbell Sleep Questionnaire.

**Table 1:** Baseline characteristics of study participants.

characteristics	Overall *n = 38*	Males *n = 16 (42*.*1%)*	Females *n = 22(57*.*9%)*
Age in years*	55 (22–83)	54.5 (22–75)	56 (25–83)
Race, n (%)			
White	18 (47.3)	9 (56.3)	9 (40.9)
Black	18 (47.3)	6 (37.5)	12 (54.5)
Other	2 (5.2)	1 (6.3)	1 (4.5)
ICU admission diagnosis, n (%)			
Sepsis (non-pulmonary)	8 (21.1)	5 (32.1)	3 (13.6)
Respiratory failure	13 (34.2)	4 (25)	9 (40.9)
Altered Mental Status	6 (15.8)	2 (12.5)	4 (18.1)
GI	2 (5.2)	1 (6.2)	1 (4.5)
Other	9 (23.7)	4 (25)	5 (22.7)

Data is count (percent), unless otherwise specified by

*median (range)

**Table 2:** Comparison of patient versus nurse Richards-Campbell Sleep Questionnaire (RCSQ) score.

	Patient (avg. score) *n = 38*	Nurse (avg. score) *n = 38*	Difference (95% CI)	P
Question 1-Sleep depth	49.7 (33.3)	44.6 (28.3)	5.1 (-3.8, 14.0)	0.25
Question 2-Sleep latency	54.6 (32.3)	51.3 (24.3)	3.3 (-7.6, 14.1)	0.55
Question 3-Awakenings	53.7 (34.2)	48.3 (25.2)	5.4 (-5.1, 15.9)	0.31
Question 4-Returning to sleep	51.8 (32.4)	54.6 (28.7)	-2.8 (-14.1, 9.1)	0.64
Question 5-Sleep quality	50.8 (31.0)	49.7 (28.7)	1.1 (-7.6, 9.8)	0.78
Total Score (AVERAGE ITEMS 1–5)	52.1 (24.9)	49.7 (23.1)	2.4 (-4.9, 9.7)	0.51

The intraclass correlation coefficients (ICC) between patient and nursing surveys’ responses are listed in Table 3. Overall, the ICC was 0.57 (95%CI 0.35, 0.76). When analyzed by sex there was an excellent intraclass correlation for men and a poor intraclass correlation for women. However, these measures were not statistically significant different. When analyzed by race, there was a fair intraclass correlation for blacks, and a good intraclass correlation for non-blacks. Related to their age, the respondents were grouped into 50 years or older versus younger than 50 years old. The ICC indicated fair intraclass correlation for both age groups. Similarly, the responders were divided into groups, with or without respiratory failure as the reason for admission. There was a fair intraclass correlation for respiratory failure group, and good intraclass for non-respiratory failure group.

**Table 3:** Cicchetti* intraclass correlation (ICC) between patient and nursing survey results.

Variable	ICC
**Overall**	0.57 (95%CI 0.35, 0.76)
**Sex**	
Male	0.84 (95%CI 0.65, 0.94)
Female	0.25 (95%CI 0.04, 0.73)
**Race**	
Black	0.46 (95%CI 0.17, 0.79)
Non-Black	0.67 (95%CI 0.40, 0.86)
**Age**	
Less than 50 years old	0.44 (95%CI 0.12, 0.82)
More than 50 years old	0.57 (95%CI 0.31, 0.80)
**Reason for Admission**	
Respiratory	0.43 (95%CI 0.11, 0.82)
Non-Respiratory	0.66 (95%CI 0.43, 0.84)

* Cicchetti (1994) gives the following interpretation for ICC: Less than 0.40—poor; Between 0.40 and 0.59—Fair; Between 0.60 and 0.74—Good; and Between 0.75 and 1.00—Excellent.

## Conclusions

We evaluated a total of 38 paired patient-nurse using the Richards-Campbell Sleep Questionnaire surveys. Similar to other published studies, our patients reported poor sleep quality with a RCSQ score of 52.1 [9, 5, 10, 11, 14], but there was a wide range of scores (Table 2 and S2 Fig). For example, Frisk et al 2003, reported a total RCSQ score of 45.5 while Richards et al 2000 reported a total score of 60. Our results also confirmed earlier findings in that patients reported falling asleep easily but that their sleep tended to be light [10, 11, 14, 8]. This was evidenced by a higher patient sleep latency score and a low sleep depth score by both patients and nurses. Patients reported a total score of 49.7 with regards to sleep depth compared to 54.6 for sleep latency.

There have been conflicting results regarding the patient-nurse reliability of accuracy of the RCSQ. Several studies have found a favorable correlation [12, 10] while others have found a poor correlation [11, 14]. In our study, the overall intraclass correlation was fair at 0.57 (95% CI 0.35,0.76). Type of ICU settings, previous nursing training in sleep hygiene, and variable inclusion criteria may account for the difference in results. Interestingly, in our study, nurses perceived that their patients slept much worse than the patients themselves, scoring lower scores on all five questions with the exception of the patients returning to sleep. This is in contrast to other published studies, whereby nurses tended to overestimate the quality of the patients’ sleep [11]. One explanation for this might be that because our nurses had not gotten any prior training in sleep hygiene, as opposed to the study conducted by Kamdar et al, 2012 in which a the surveys were done during a preexisting MICU sleep quality improvement project.

Our study is the first to show how patient related factors may influence nursing perception of sleep quality in the medical ICU. Frisk et al 2003 investigated how patient age, length of stay in the ICU as well as usage of hypnotics may have impacted patients perception of sleep. However, no assessment of paired patient-nurse was made. Our hypothesis was that age, race, reason for admission and sex will influence sleep perception by both patients and nurses. We found that when assessed by age, there was a fair intraclass correlation amongst the age groups. When race is a factor, a good intraclass correlation existed for non-black patients (0.67, 95%CI 0.40, 0.86) compared to a fair intraclass assessment for black race (0.46, 95%CI 0.17, 0.79), but this was not statistically significant. A recent meta-analysis looking at normal sleep in African-Americans and white found that African Americans had worse objective and subjective sleep, with poorer sleep continuity, duration and less slow wave sleep (i.e. deep sleep) [15]. It is therefore not surprising that the intraclass correlation was slightly worse for this category. When examining intraclass correlation by reason for admission to the ICU, a good correlation was found if the patients were admitted for non-respiratory reasons (0.66, 95%CI 0.43, 0.84) compared to those with respiratory illness (0.43, 95%CI 0.11, 0.82), in which there was a fair correlation. This result can be explained by the fact that patients with respiratory illness report worse sleep quality with changes in sleep architecture and sleep fragmentation [16].

The most striking discordance between patient and nursing perception of sleep was discovered when assessing the intraclass corrrelation by sex. There was poor intraclass correlation for women (0.25, 95%CI 0.04, 0.73) compared to an excellent correlation for men (0.84, 95%CI 0.65, 0.94). This was not statistically significant likely due to small sample size. It is well established that a paradox exists between women’s objective and subjective sleep quality. Numerous studies have found that healthy women objectively have better quality sleep as measured by PSG, but consistently report poorer quality and more disrupted sleep across various stages of life when compared to men [6, 17, 18, 19]. In addition, when compared to men, women have a higher risk of insomnia (risk ratio, 1.41; 95% CI, 1.28–1.55) [20]. It therefore not unexpected that women would report poor sleep on the RCSQ compared to men and that the corresponding nursing perception would reflect this.

There are a number of potential limitations of our study. First, although we had a diverse sample, the study size was small. This may explain why we were not able to reach statistical significance. Second, the study was performed at a single center. This may limit the generalizability of the results. Nevertheless, we believe that our findings can be utilized to further assess the sub-groups we identified as having significantly different results. Third, this was a retrospective study. Still, our results add to the body of evidence that this subject is in need of larger prospective studies to evaluate different sub-groups and interventions.

In conclusion, the results of our study confirm that patients in our ICU have poor sleep and that nursing perception reflects this with a fair intraclass correlation. However, the aggregate results do not allow us to determine if there are patient related factors that might influence the results. When examined by patient related factor, the greatest divergence between patient and nursing perception of sleep in the ICU using the RCSQ was patient female sex. Less variable intraclass correlation was noted for race and reason for admission. In light of these results, discernment should be used when interpreting the intra-rater reliability of the RCSQ in the ICU and provide an educational opportunity to improve sleep quality in the units that is more personalized and patient-centered.

## Supporting information

S1 Copy of Data No HIPPA submission. (XLSX)

S1 Fig Flow diagram of patient and nursing surveys. (TIF)

S2 Fig Scatter-plot of paired patient-nursing total score from the Richards-Campbell Sleep Questionnaire surveys. (TIF)

## Abbreviations

ICC: Intraclass correlation
ICU: Intensive Care Unit
RCSQ: Richards-Campbell Sleep Questionnaire
