# Supplementary figures and images for: Patient-related factors may influence nursing perception of sleep in the Intensive Care Unit
Source: PLoS One. 2020 Jan 6;15(1):e0226323. doi: 10.1371/journal.pone.0226323 (PMC6944385; doi:10.1371/journal.pone.0226323)

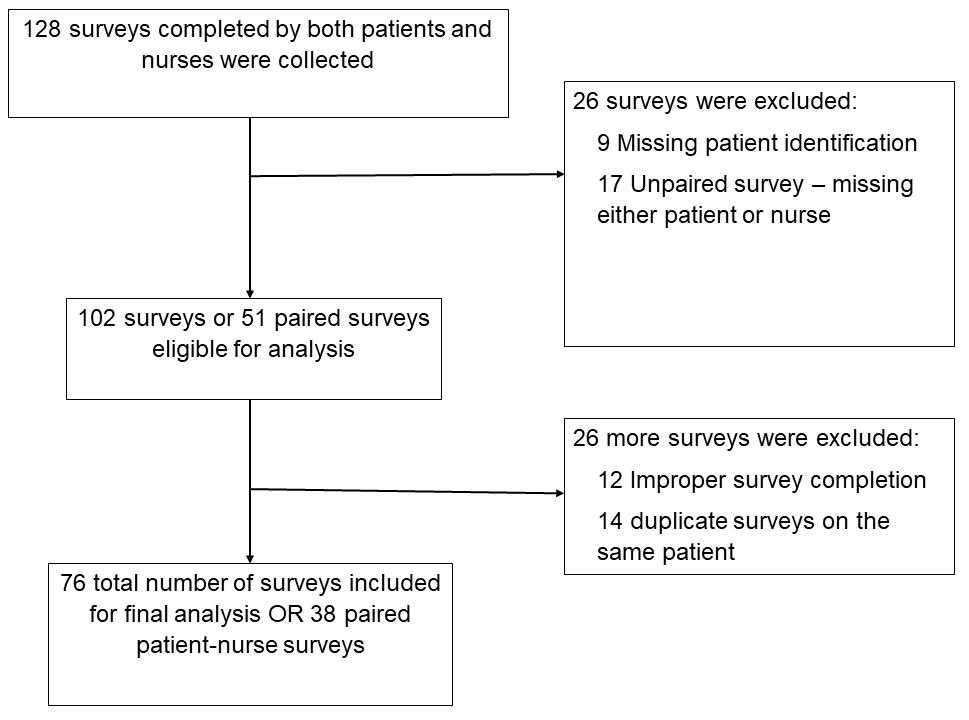

Supplement: S1 Fig — (TIF) [file pone.0226323.s002.tif]

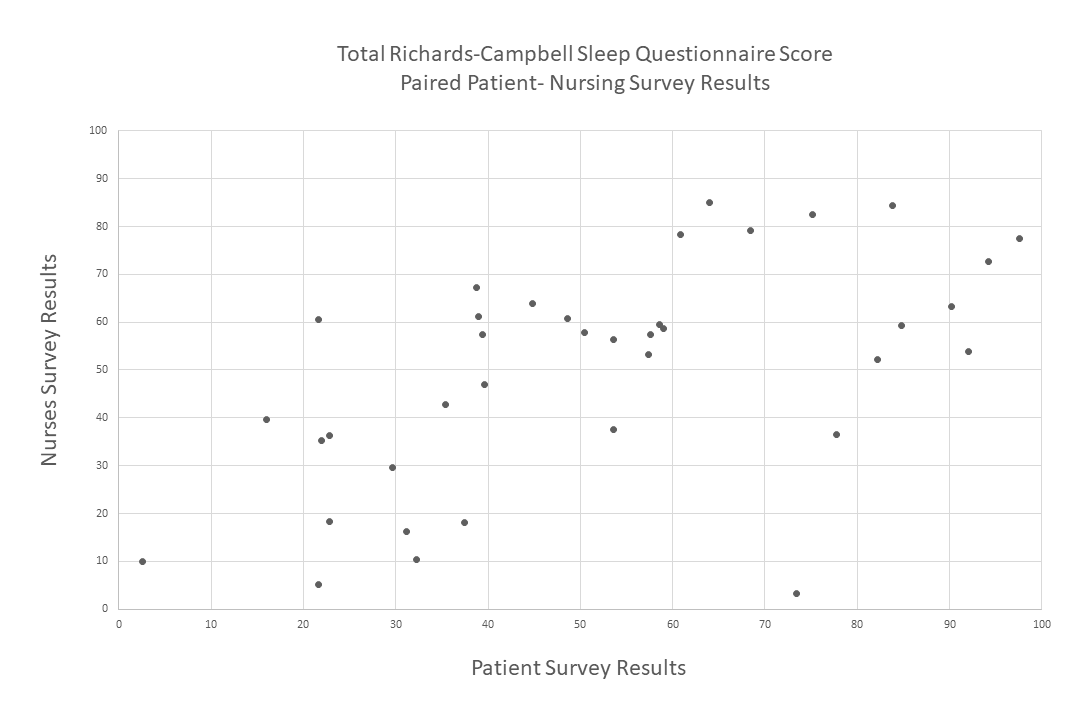

Supplement: S2 Fig — (TIF) [file pone.0226323.s003.tif]
